# Electric Resistance and Curing Temperature Development of Carbon Fiber-Reinforced Conductive Concrete: A Comparative Study

**DOI:** 10.3390/ma17164045

**Published:** 2024-08-14

**Authors:** Lei Zhang, Siyuan Chen, Weichen Tian, Yuan Tang, Qiang Fu, Ruisen Li, Wei Wang

**Affiliations:** 1 School of Water Conservancy and Civil Engineering, Northeast Agricultural University, Harbin 150030, China; 2 School of Civil Engineering, Harbin Institute of Technology, Harbin 150090, China; 3 School of Infrastructure Engineering, Nanchang University, Nanchang 330031, China; 4 Aerospace Science and Industry Defense Technology Research and Testing Center, Beijing 100854, China

**Keywords:** ohmic heating curing, conductive concrete, electric resistance, curing temperature

## Abstract

The development of electric resistance is a key factor affecting the performance of conductive concrete, especially the electrical–thermal performance. In this work, the effects of different influencing factors (including the water-to-binder ratio, coarse aggregate content and carbon fiber (CF) content) on the electric resistance of conductive concrete were systematically investigated. At the same time, ohmic heating (OH) curing was applied to fabricate CF-reinforced conductive concrete (CFRCC) under a negative temperature environment at −20 °C. The effects of different factors on the electrothermal properties (curing temperature and conductive stability) of the samples were studied. The mechanical strengths of the CFRCC cured by different curing conditions were also tested, and the feasibility of OH curing for preparing CFRCC in a negative-temperature environment was verified at various electric powers. This work aims to give new insights into the effects of multiple factors on the performance of CFRCC for improved concrete construction in winter.

## 1. Introduction

Concrete is the most widely used construction material in the world, because it is easy to construct with low economic cost [1,2,3,4]. However, concrete construction in cold regions is still a severe issue, which largely impedes infrastructure construction and economic development in these areas, which occupy a large part of the land area [5,6,7]. To solve this problem, many methods have been presented to fabricate concrete in negative-temperature environments. In general, two kinds of strategies are applied: On the one hand, a positive-temperature environment can be created to achieve the ongoing curing process for concrete structures, and a simple temporary warm shed has been established to accommodate the concrete structure [8,9,10]. On the other hand, the ice point of the water inside the concrete was lowered with the aid of chemical admixtures to prevent the water inside the structure from freezing [11,12]. However, the application of the above strategies is significantly restricted by the environmental conditions. To be specific, when the environmental temperature is below −15 °C, both of the strategies fail due to the difficulty of building the warm shed and the inability of the chemical admixture to work [13,14]. Additionally, existing winter concrete construction methods face various risks, including (1) difficult human operation in cold environments that increases the uncertainty; (2) the properties of the raw materials may suffer damage during the transportation process; and (3) the early-age curing of concrete may be easily influenced due to the harsh external environment, resulting in the low quality of the concrete [15]. In this context, a novel ohmic heating (OH) curing method was introduced to fabricate concrete structures in actual cold environments with simple device support [16,17].

To be more precise, the heat generated from the electric current passing through the conductive phases is used to increase the curing temperature for OH-cured samples [18,19,20,21]. It should be noted that the main conductive phases in pure concrete are only pore solutions [22,23,24] and the OH curing process is not sustainable for pure concrete because the solution is quickly consumed, especially in negative-temperature environments [25,26]. Therefore, the electrical conductivity of concrete should be enhanced with the help of additional materials to ensure stable electrical conduction inside the concrete [27,28,29,30], which was the basis for the implementation of OH curing. In previous studies, conductive fibers including carbon fibers (CFs), carbon black, steel fibers, stainless wire, nano carbon fibers (NCFs), and graphene, etc. showed a good ability to reduce the electric resistance of concrete [31,32,33,34,35,36]. Among these materials, CFs with a larger volumetric size exhibited a much better effect in terms of enhancing the electrical conductivity of the samples because it was reported to be easier to construct the macroscopic connected network inside the sample [37,38,39]. Although CFs are promising for improving the electrical conductivity of concrete, it is still important to clarify the effects of different factors on the electrical conductivity of concrete to ensure the ongoing OH curing process. In previous studies, the specific effect of CFs on the electrical conductivity of OH-cured samples has been disclosed [40], while the regular development of certain parameters in OH-cured concrete has still not been investigated, and factors including the water-to-binder ratio, CF content, and coarse aggregate content could affect the electrical conductivity of the concrete [41,42,43,44]. To be specific, the water-to-binder ratio could directly influence the amount of conductive pore solution in the sample, and the inclusion of coarse aggregate could significantly influence the performance of the sample, because this would change the packing density and affect the distribution of the CFs.

To better clarify the effect of different factors on regular parameter development in CF-reinforced conductive concrete (CFRCC), the specific effects of the water-to-cement ratio, coarse aggregate content, and CF content were investigated under room temperature (RT) curing and OH curing conditions. It needs to be noted that some experiments were only conducted under RT curing to obtain the preliminary results as the basis for further OH curing. To be precise, the specific effects of the water-to-cement ratio and coarse aggregate content were verified under RT curing conditions, and the experiments regarding the effects of the CF content were conducted under both RT and OH curing conditions. The electric resistance, compressive strength, and curing temperature were chosen as the indicators to determine the various effects of the above parameters on the electric resistance or heating performance in CFRCC. Once the optimal material composition was determined, the CFRCC was subjected to OH curing with various electric powers under −20 °C, aiming to clarify the different curing conditions created by OH curing to further confirm the feasibility for this novel curing method. This work provides foundational knowledge of the electro-thermo parameters of conductive concrete.

## 2. Materials and Methods

### 2.1. Raw Materials

P.O. 42.5 cement was used as the main cementitious material, and river sand with a fineness modulus of 2 was used as the finer aggregate; the diameter of the coarse aggregate was within 5–20 mm. Chopped CFs produced by Tokyo Toray (Tokyo, Japan) with a length of 4 mm were used to improve the electrical conductivity of the sample. A polycarboxylic acid-based superplasticizer (SP) purchased from HIT QiangShi (Harbin, China) was used to modify the workability of the fresh mixture.

### 2.2. Mix Proportions and Sample Preparation

**Figure 1:**
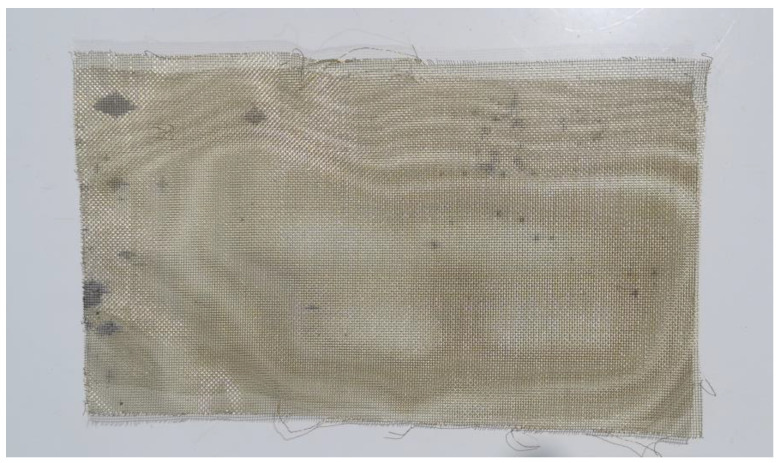
Image of copper mesh.

To investigate the effects of various factors on the performance of CFRCC, the water-to-cement ratios were varied from 0.2 to 0.4 with 0.05 as the step, the CF content was changed within the range of 0–1.0 vol% with 0.25% as the increase step, and the coarse aggregate was used to replace the paste in a volume from 0 to 40% with 10% as the step. The specific preparation of CFRCC is as below: the cement and CFs were slowly mixed in a stirring pot for 3 min, the fine aggregate and coarse aggregate were then mixed for 3 min, then all the dry materials were put together in the stirring pot for another 3 min of stirring. Finally, the solution of water and SP was poured into the pot to further stir for 3 min, and the fresh mixture was cast into cubic molds with a side length of 100 mm. Two copper meshes with the dimensions 100 mm × 120 mm were inserted into the mixture with a distance of 95 mm, to ensure the ongoing OH curing process. The image of copper mesh is depicted in Figure 1.

### 2.3. Curing Regimes

#### 2.3.1. RT Curing Procedure

The temperature was set to be 20 ± 2 °C and the humidity was controlled to be higher than 95% for the RT curing regime, and the curing durations for RT curing were varied based on the specific requirements.

#### 2.3.2. OH Curing Procedure

The fresh mixture was immediately put into the refrigerator after casting into the molds, the environment temperature in the refrigerator was set to be −20 °C, and the electric power of OH curing was set to be constant at 30 W during the OH curing process. The electric voltage applied to the sample was adjusted based on Equation (1) to keep the electric power constant during the OH curing procedure.

(1) $$P=\frac{U^{2}}{R}$$

in which *P* means the electric power (W), *U* represents the electric voltage (V), and *R* indicates the measured electric resistance (Ω).

### 2.4. Electrical Performance Evaluation

#### 2.4.1. Electric Resistance Measurement

The two-electrode method was used to measure the electric resistance of the sample through the electrodes. The test frequency was set to be 10 kHz; it was reported by Han et al. that this frequency could eliminate interface resistance [45].

#### 2.4.2. Electric Resistance Development Factor (ERF)

The absolute value of electric resistance in the sample is not significant, and the electric resistance change regularity is more important, because it can reflect the electrical conductivity development law inside the sample, which is strongly connected to the sustainable OH curing process. Thus, the electric resistance development factor (*ERF*) is presented in this work, and is shown in Equation (2).

(2) $$\mathrm{ERF}=\frac{R_{i}}{R_{0}}$$

in which *R*_i_ represents the electric resistance of CFRCC at time point i and *R*_0_ indicates the initial electric resistance of fresh CFRCC.

### 2.5. Curing Temperature Measurement

**Figure 2:**
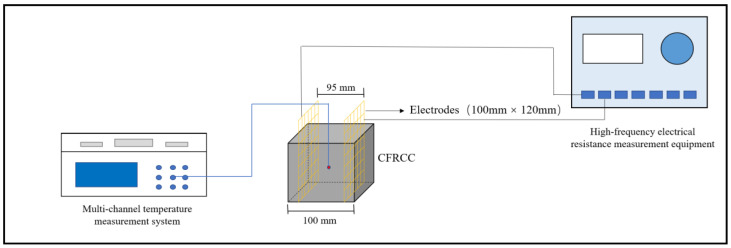
OH curing diagram.

The curing temperature was measured by the thermocouple embedded at the center of the sample, and the multi-channel temperature record system was used to record the temperature development during the OH curing process. The OH curing diagram is depicted in Figure 2. Moreover, it should be emphasized that because the concrete was transferred from the mixing process (room temperature condition) to the negative-temperature environment, and the OH curing was immediately conducted, there showed no possibility for the sample to suffer from freezing.

### 2.6. Strength Test

The compressive strengths of CFRCC were tested at various curing durations based on the specific experiment requirement. The test was conducted according to ASTM C109/C109M-11 [46], and the loading rate was set to be 2.4 kN/s during the test process.

## 3. Results and Discussion

### 3.1. Effect of Water-to-Cement Ratio on CFRCC

In this section, the basic effect of the water-to-cement ratio on the performance (including the initial electric resistance and compressive strength) was clarified. It should be noted that RT curing was conducted in this section as the foundation.

#### 3.1.1. Effect of Water-to-Cement Ratio on Initial Electric Resistance

**Figure 3:**
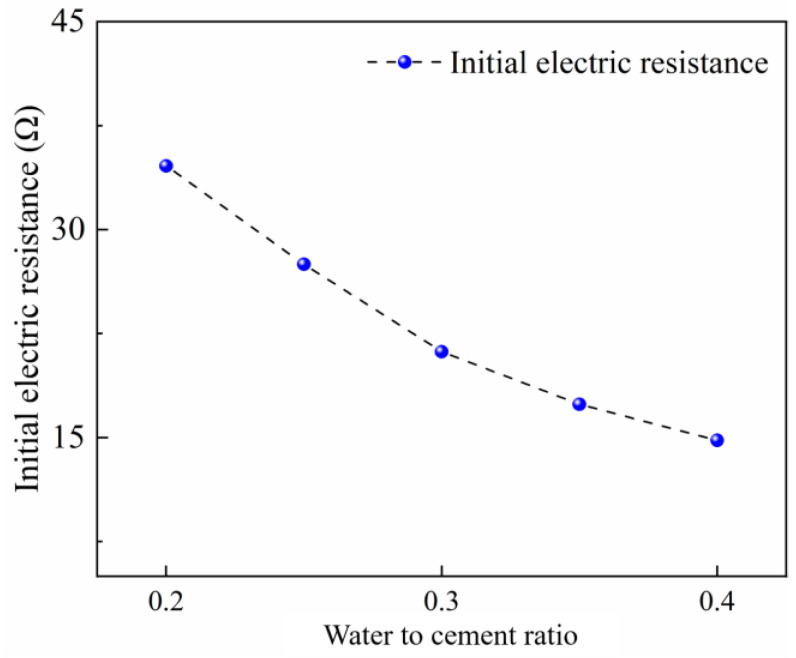
Effect of water-to-cement ratio on initial electric resistance of CFRCC.

The initial electric resistance of CFRCC with various water-to-cement ratios is depicted in Figure 3 to obtain the preliminary results. In this experiment, the CF content was pre-fixed to be 0.75 vol% based on previous test results [47] and the coarse aggregate content was 20% of the volume of the paste. It can be found from the test results that the increasing water content could improve the conductivity of the samples due to the content of the conductive pore solution being much enlarged with a higher water-to-cement ratio, especially at the early curing stage; the pore solution in the fresh concrete was not consumed. Moreover, it should be noted that all of the initial electric resistance values in the CFRCC with water-to-cement ratios of 0.2 to 0.4 were lower than 35 Ω, and such low values satisfied the requirement for further OH curing.

#### 3.1.2. Effect of Water-to-Cement Ratio on Compressive Strength

**Figure 4:**
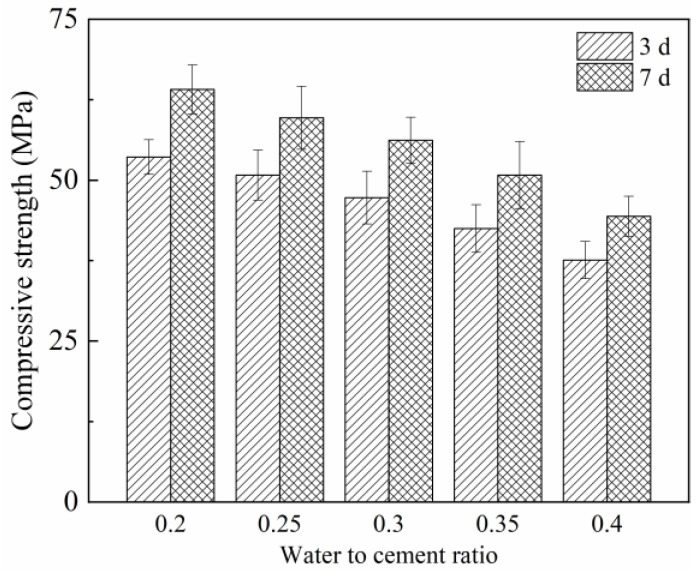
Effect of water-to-cement ratio on compressive strength of RT-cured CFRCC at 3 days and 7 days.

The compressive strengths of the RT-cured CFRCC with various water-to-binder ratios were tested at different curing ages (3 days and 7 days) and are shown in Figure 4. It can be found that the compressive strengths for the samples showed a gradual reduction trend with the increased water-to-cement ratio. For the CFRCC with the water-to-cement ratio of 0.3, the 3 days compressive strength was near 50 MPa and the 7 days compressive strength reached up to 60 MPa, which was suitable for the early age strength formation of CFRCC in a negative-temperature environment; thus, the water-to-cement ratio was set to be 0.3 in the following experiments.

### 3.2. Effect of Coarse Aggregate Content on Electric Resistance

**Figure 5:**
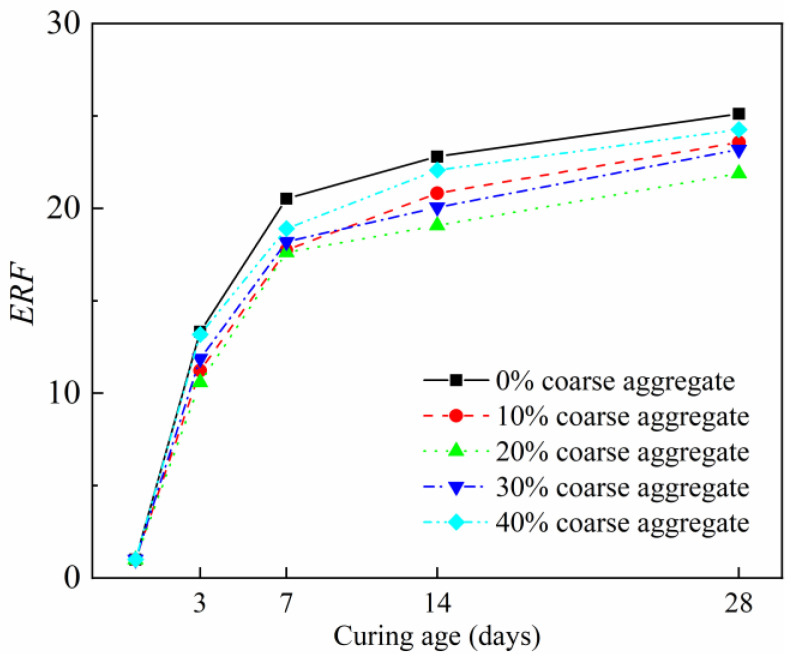
Effect of coarse aggregate content on *ERF* of CFRCC.

In this section, the specific effect of the coarse aggregate content on the electric resistance of the CFRCC was clarified. The samples were cured under RT curing conditions. The aggregate contents were varied from 0 to 40% of the volume of the mortar, and the CF content was fixed to be 0.75 vol%. Figure 5 exhibits the *ERF* of the CFRCC at different RT curing ages (0 day, 3 days, 7 days, 14 days, and 28 days), and it can be seen that the inclusion of coarse aggregate was advantageous to reducing the *ERF* of the CFRCC. Generally, the most significant increase in the ERF could be found from 0 d to 3 d in the samples due to the intense hydration reaction at the early curing stage, that the conductive pore solution was much consumed. Moreover, after 7 days, the increased velocity of the *ERF* was lowered due to the hydration reaction becoming stable after the 7-day curing stage. In addition, the inclusion of the coarse aggregate was advantageous to improving the conductivity of the sample, and this improvement effect exhibited an increasing then decreasing trend with the increased coarse aggregate content. To be more specific, when the coarse aggregate content was 20%, the sample showed the lowest *ERF*, and this represented the most stable electrical conductivity development for the sample. And when the coarse aggregate contents exceeded 20%, the ERF of the sample gradually increased. Collectively, the coarse aggregate showed the ability to improve the electrical conductivity, which was related to the modification effect of the coarse aggregate on altering the CF distribution situation in the CFRCC, because the much larger volumetric size of the coarse aggregate would much more easily influence the CFs inside the sample.

### 3.3. Effect of Coarse Aggregate Content on Compressive Strength

**Figure 6:**
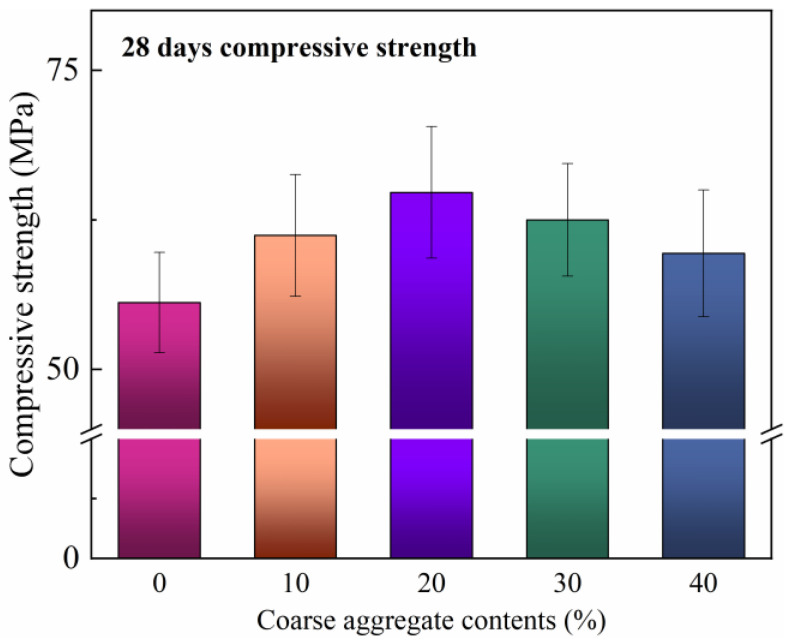
Effect of coarse aggregate content on compressive strength of CFRCC.

The compressive strengths of the 28-day RT-cured CFRCC incorporating various coarse aggregate contents are depicted in Figure 6. It can be found that the inclusion of coarse aggregate could enlarge the mechanical strengths of the samples, and that the samples with aggregate all showed higher 28 days compressive strengths than those of the pure mortar samples. This could be explained by the fact that under this material system, the incorporation of coarse aggregate improved the degree of compact packing of the particles inside the samples. To be more precise, the CFRCC containing 10–40% coarse aggregate showed, respectively, a strength increase of 11.6%, 17.8%, 13.6%, and 8.2%. The 20% coarse aggregate content was verified by the mechanical strength test results. Thus, the optimal content of coarse aggregate added to the CFRCC was determined to be 20% according to both the electric and strength test results.

### 3.4. Effect of CF Content on Initial Electric Resistance

**Figure 7:**
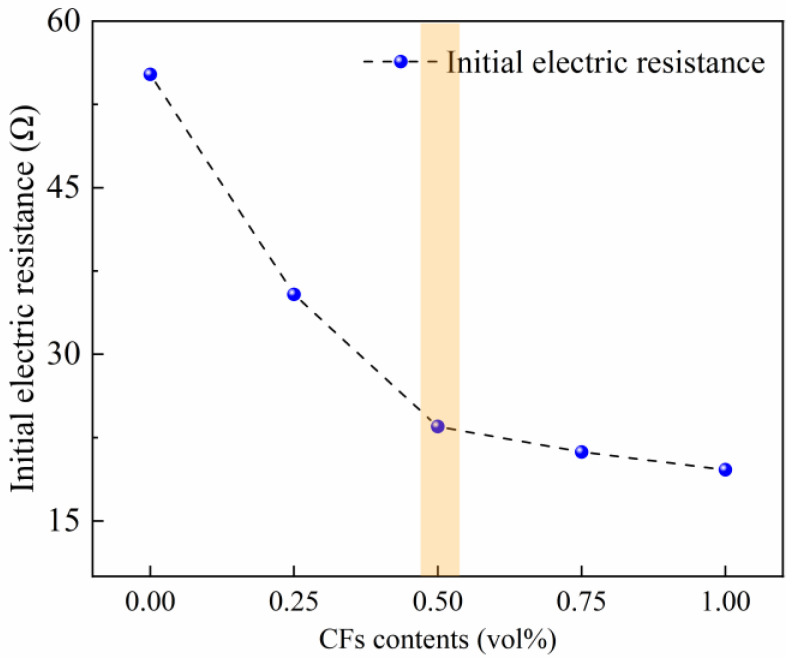
Effect of CF content on initial electric resistance of CFRCC.

The initial electric resistance of CFRCC containing various CF contents is depicted in Figure 7 to reflect the effect of CF addition on the CFRCC, and the preliminary results were further verified during the OH curing process. With the addition of the CFs, the initial electric resistance was much reduced from 55.2 Ω to 19.6 Ω with the CF content varying from 0 to 1.0 vol%. This was because the addition of CFs created more conductive paths inside the sample, which caused a more significant effect than the conductive pore solution alone on the electrical conductivity of the CFRCC. The specific effect of the CFs in the further OH curing process will be clarified in the following section.

### 3.5. Effect of CF Content on OH Curing Process

#### 3.5.1. ERF Change

**Figure 8:**
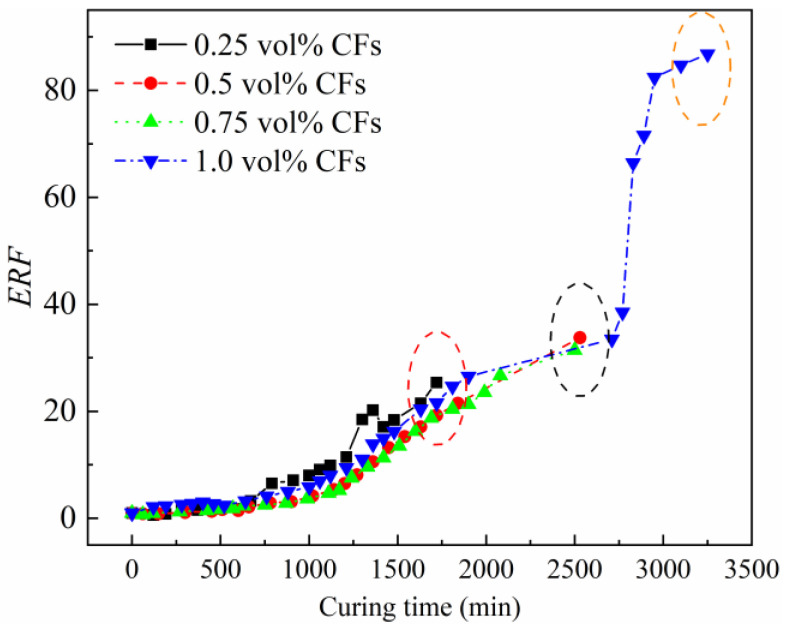
Effect of CF content on *ERF* of OH-cured CFRCC.

In this section, the CFRCCs with various CF contents were subjected to OH curing with an electric power of 30 W with an environmental temperature of −20 °C. The samples containing 0.25 vol%–1.0 vol% CF contents were used for comparison, because the samples with no CFs could not undergo the OH curing process even for a short time [23]. It can be found that most of the time, the increase in the CF content could lower the *ERF* of the sample during the OH curing process, as depicted in Figure 8, indicating the more stable conductive network inside these samples, which is beneficial for prolonging the curing duration of OH-cured CFRCC, thus improving the curing efficiency of the concrete fabricated under negative-temperature environments. To be more specific, the sample with 0.25 vol% CFs showed the highest *ERF* compared with those of the samples with higher CF contents, and when the CF content reached 0.5 vol%, the sample exhibited a comparable *ERF* increasing trend with the incorporation of 0.75 vol% CFs, indicating the beneficial effect of a 0.5 vol% CF content on improving the electrical conductivity of the samples. However, when the CF content reached 1.0 vol%, a prolonged curing duration was observed for the sample, while the ERP was larger than those of other samples, indicating that after reaching the percolation threshold, the increase in the CF contents exhibited an insignificant effect.

In addition, the curing durations of the different samples showed an obvious difference, and the sample with 1.0 vol% CFs showed the longest curing duration. The increased conductive network inside the sample could result in this prolonged curing duration; however, the performance should be verified further to clarify the effect of the prolonged curing duration.

#### 3.5.2. Curing Temperature Development

**Figure 9:**
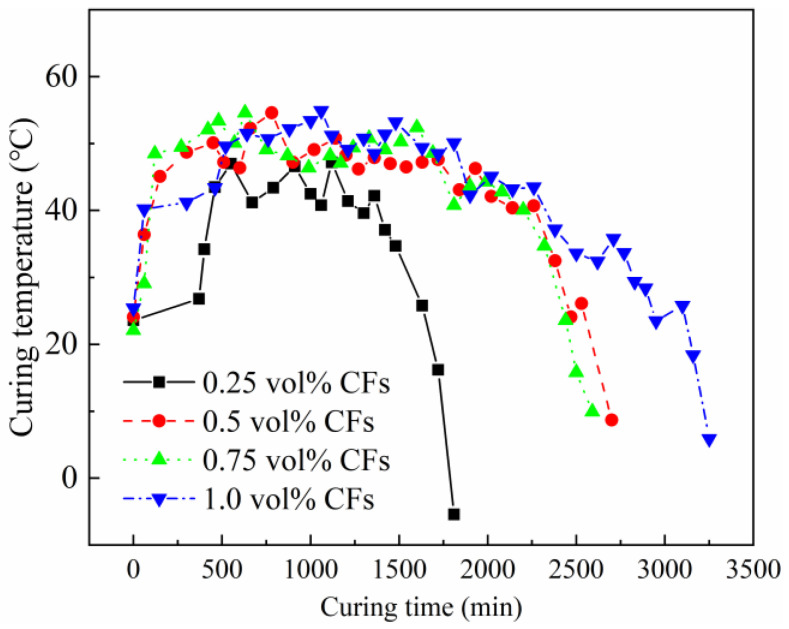
Effect of CFs on curing temperature of OH-cured CFRCC.

The curing temperatures of the OH-cured CFRCC with various CF contents are shown in Figure 9, and the curing temperature could reflect the curing duration of the samples, which are disclosed in Section 3.5.1. It is depicted in Figure 9 that the lower CF contents were not suitable for the ongoing curing process of the CFRCC in a negative-temperature environment. It can be found that when the CF content was 0.25 vol%, the curing temperature of the sample was mainly concentrated in the range 40–50 °C. A significantly decreasing trend was seen at around 1250 min, and the temperature became negative at around 1750 min, which could not satisfy the OH curing requirement. For the samples containing 0.5 vol% and 0.75 vol% CF contents, the curing durations were prolonged to 2750 min, with the main curing temperature within the range 40–55 °C. The longer curing duration and higher curing temperature ensured the strength development of the CFRCC in a negative-temperature environment. Moreover, for the samples containing 1.0 vol% CFs, the curing temperature was similar to that of the samples containing 0.5 vol% and 0.75 vol% CFs, while the curing duration was prolonged to 3250 min due to the larger amount of conductive paths inside the sample. Based on the results of the ERF development and curing temperature results, the compressive strengths were tested.

#### 3.5.3. Strength Development

**Figure 10:**
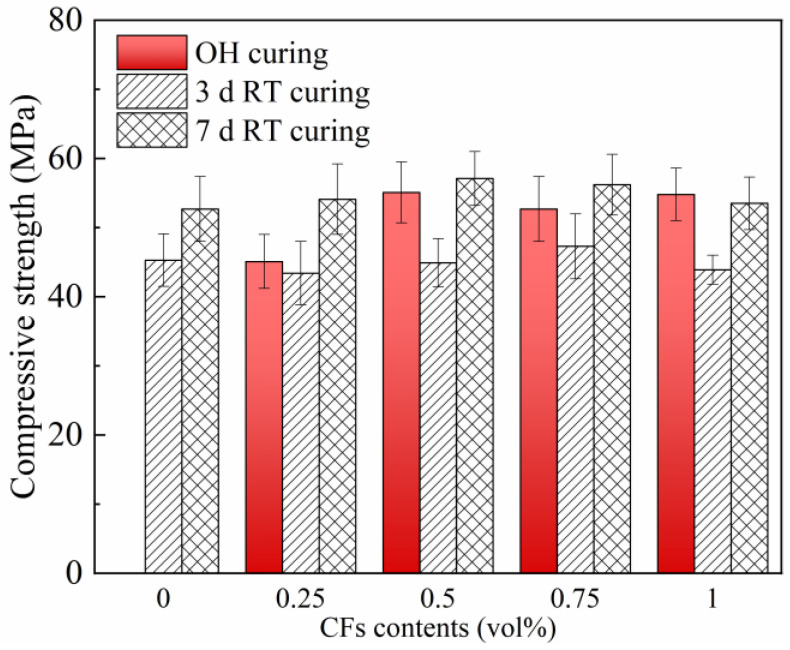
Effect of CF content on compressive strength of OH-cured samples.

The compressive strengths the OH-cured CFRCC with various CF contents were tested and compared with those of the samples subjected to RT curing for 3 days and 7 days, respectively. The curing durations for the samples were consistent with the curing temperature results. To be specific, the CFRCC samples with a 0.25 vol% CF content were cured by 30 W OH curing for 1750 min, those with 0.5 vol% and 0.75 vol% CFs were cured for 2750 min, and the 1.0 vol% CF sample was cured by OH curing for 3250 min. The mechanical strengths are shown in Figure 10, and it can be found that OH curing could improve the compressive strength of CFRCC at −20 °C; the OH-cured samples always exhibited higher strengths to those of the 3-day RT-cured ones, even with a much shorter curing duration. Specifically, for the sample with a 0.5 vol% CF content, the compressive strength could reach up to 52 MPa, which was comparable to that cured by 7 d of RT curing. Moreover, the compressive strengths for the samples containing 0.5–1.0 vol% CF were similar under the same curing regime, and based on this, the optimal CF content could be determined to be 0.5 vol%.

### 3.6. Effect of Electric Power on OH-Cured CFRCC

**Figure 11:**
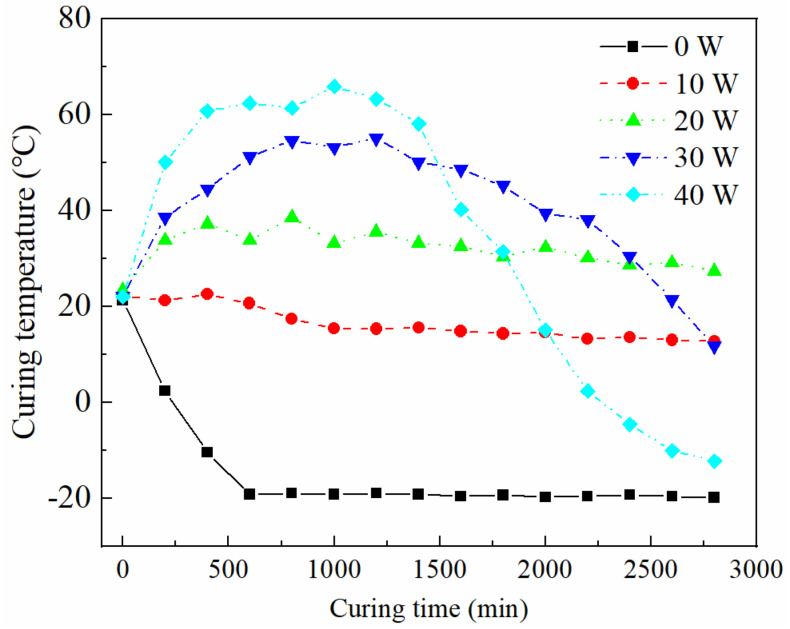
Effect of electric power on curing temperatures of OH-cured CFRCC.

The optimal water-to-cement ratio, coarse aggregate content, and CF content were determined based on the preliminary experiments in the above sections, and the CFRCC was subjected to OH curing with the optimal determined material composition. To be specific, during the OH curing process, five electric power regimes were applied to the CFRCC in a −20 °C negative temperature environment, and the curing temperatures were recorded as shown in Figure 11. In general, a higher electric power could increase the curing temperature of the OH-cured samples because the electric power and curing temperature were highly connected based on the basic heat transfer theory that the electric-induced heat would increase to a high temperature in the OH-cured CFRCC. To be specific, the main curing temperatures were concentrated around −20 °C, 20 °C, 35 °C, and 50 °C for the OH-cured samples with the respective electric powers of 0 W, 10 W, 20 W, and 30 W, exhibiting an obvious increasing trend. However, a higher electric power of 40 W would increase the highest curing temperature of the sample (>60 °C) and reduce the high-temperature curing duration for the OH-cured sample, which may be related to the conductive network inside the CFRCC being destroyed by the high electric power, resulting in an unsustainable OH curing process. Under this circumstance, high-electric-power OH curing regimes should be conducted only when the mix proportion of the sample is optimized to improve the curing quality of the sample in harsh environments.

## 4. Conclusions

In this work, the effects of the water-to-cement ratio, CF content, and coarse aggregate content on the electric resistance, curing temperature, and compressive strength of CFRCC were systematically investigated, with the aim of disclosing the basic parameter effect on the electro-thermo properties and strength of the CFRCC. The conclusions are highlighted below.

(1) The increase in the water-to-cement ratio could improve the electrical conductivity of the CFRCC due to the increased conductive pore solution inside the sample, but could be harmful to the mechanical strength of the CFRCC.
(2) The inclusion of coarse aggregate was beneficial for the electrical conductivity because it could modify the CF distribution inside the CFRCC sample. When the coarse aggregate content was lower than 20%, the increase in the coarse aggregate content could enhance the compressive strength of the CFRCC.
(3) CFs could effectively modify the electrical conductivity of the CFRCC and could prolong the OH curing duration in a negative-temperature environment because of the much more stable conductive network, which increased the strength of the OH-cured concrete structure at a feasible curing temperature.
(4) The electric power showed a strong relationship with the curing temperature based on the basic heat transfer theory. A higher electric power could increase the curing temperature for the OH-cured CFRCC at −20 °C, but when the electric power was too high (40 W), the OH curing process was terminated much earlier, which may be related to the destroyed conductive network inside the sample.

## Data Availability

The original contributions presented in the study are included in the article, further inquiries can be directed to the corresponding author.
